# P-1634. Socioeconomic and Demographic Factors Associated with COVID-19 Antiviral Treatment Prescription in a Large Healthcare System

**DOI:** 10.1093/ofid/ofaf695.1810

**Published:** 2026-01-11

**Authors:** Majd Alsoubani, Maureen Campion, Gabriela Andujar Vazquez

**Affiliations:** Tufts Medical Center, Boston, Massachusetts; Tufts Medical Center, Boston, Massachusetts; Tufts Medical Center, Boston, Massachusetts

## Abstract

**Background:**

COVID-19 has disproportionately impacted racial, ethnic minorities and patients with lower socioeconomic status. The introduction of antiviral treatments has altered the clinical course of disease however drug-drug interactions and route of administration have limited their use. In this study, we aim to evaluate the socioeconomic and demographic factors associated with the prescription of COVID-19 treatments in a large healthcare system in Massachusetts.

**Methods:**

This is a retrospective study including adult patients with a COVID-19 diagnosis or antiviral prescription including n/r, remdesivir and molnupiravir. The Vizient Clinical Database, which captures patient-level data was used to identify encounters between Oct/2022 and Jul/2024. The primary outcome was evaluating socioeconomic and demographic factors associated with the prescription of COVID-19 antivirals. Demographic data including Vizent vulnerability index (VVI) were collected. Higher VVI scores indicate higher social vulnerability ranging from -3 to 3. Patient characteristics by treatment were presented as percentages for categorical variables and medians with interquartile ranges for continuous variables. The primary analysis was logistic regression evaluating factors associated antiviral use.

**Results:**

We identified 10,648 patients who received antivirals or had a diagnosis of COVID-19 of which 3498 (32.9%) received antivirals. The median VVI in our cohort was low indicating overall less vulnerable population –0.75 (IQR -1.11, -0.03). Patients who received treatment were more likely to be older and male. Moreover, English speaking patients were less likely to be prescribed anti-viral treatment (Table 1). In the multivariable model, older age, inpatient encounter and non-White race remained significantly associated with increased odds of COVID-19 treatment (Table 2). Outpatient encounter was associated with reduced odds of getting antivirals as compared to patients seen in the ED.

**Conclusion:**

In this large retrospective study, we found several factors that significantly influenced the likelihood of receiving antivirals. Despite a generally low vulnerability index in the study population, disparities in treatment based on race and encounter highlight challenges in COVID-19 care delivery.

**Disclosures:**

All Authors: No reported disclosures

**Table 1:**
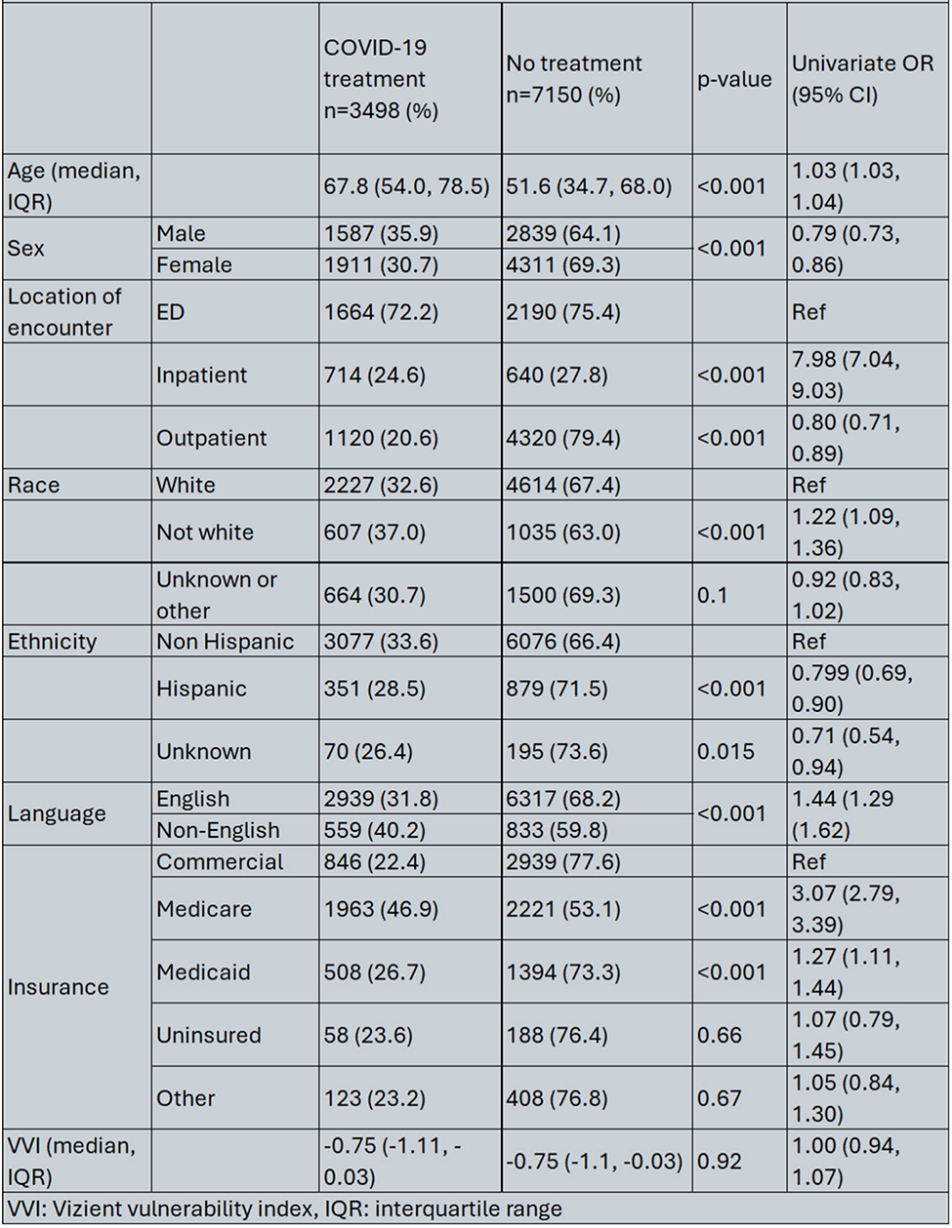
Baseline demographic and clinical characteristics of patients by treatment

**Table 2:**
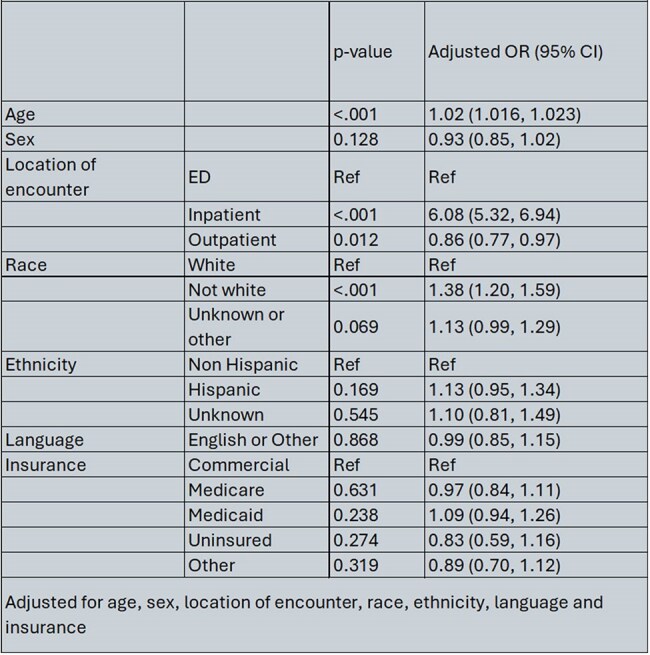
Adjusted logistic regression model of patients who received COVID-19 treatment compared to no treatment

